# Genetic diversity of *Bartonella* species in small mammals in the Qaidam Basin, western China

**DOI:** 10.1038/s41598-021-81508-w

**Published:** 2021-01-18

**Authors:** Huaxiang Rao, Shoujiang Li, Liang Lu, Rong Wang, Xiuping Song, Kai Sun, Yan Shi, Dongmei Li, Juan Yu

**Affiliations:** 1 Department of Public Health and Preventive Medicine, Changzhi Medical College, Changzhi, 046000 China; 2 Department of Basic Medical Sciences, Changzhi Medical College, Changzhi, 046000 China; 3 Institute for Communicable Disease Control and Prevention, Qinghai Center for Disease Control and Prevention, Xining, 810007 China; 4 State Key Laboratory for Infectious Disease Prevention and Control, Collaborative Innovation Center for Diagnosis and Treatment of Infectious Diseases, National Institute for Communicable Disease Control and Prevention, Chinese Center for Disease Control and Prevention, Beijing, 102206 China; 5 Wulan Center for Disease Control and Prevention, Wulan, 817100 China

**Keywords:** Bacterial genetics, Epidemiology

## Abstract

Investigation of the prevalence and diversity of *Bartonella* infections in small mammals in the Qaidam Basin, western China, could provide a scientific basis for the control and prevention of *Bartonella* infections in humans. Accordingly, in this study, small mammals were captured using snap traps in Wulan County and Ge’ermu City, Qaidam Basin, China. Spleen and brain tissues were collected and cultured to isolate *Bartonella* strains. The suspected positive colonies were detected with polymerase chain reaction amplification and sequencing of *gltA*, *ftsZ*, RNA polymerase beta subunit (*rpoB*) and *ribC* genes. Among 101 small mammals, 39 were positive for *Bartonella*, with the infection rate of 38.61%. The infection rate in different tissues (spleens and brains) (*χ*^2^ = 0.112, *P* = 0.738) and gender (*χ*^2^ = 1.927, *P* = 0.165) of small mammals did not have statistical difference, but that in different habitats had statistical difference (*χ*^2^ = 10.361, *P* = 0.016). Through genetic evolution analysis, 40 *Bartonella* strains were identified (two different *Bartonella* species were detected in one small mammal), including *B. grahamii* (30), *B. jaculi* (3), *B. krasnovii* (3) and Candidatus *B. gerbillinarum* (4), which showed rodent-specific characteristics. *B. grahamii* was the dominant epidemic strain (accounted for 75.0%). Furthermore, phylogenetic analysis showed that *B. grahamii* in the Qaidam Basin, might be close to the strains isolated from Japan and China*.* Overall, we observed a high prevalence of *Bartonella* infection in small mammals in the Qaidam Basin. *B. grahamii* may cause human disease, and the pathogenicity of the others *Bartonella* species needs further study, the corresponding prevention and control measures should be taken into consideration.

## Introduction

*Bartonella* is a genus within the *Bartonellaceae* family in the Alphaproteobacteria class. *Bartonella* species are small, intracellular, vector-borne hemotropic gram-negative bacteria, some of which can infect a variety of mammals and cause human Bartonellosis^1^. The *Bartonella* species and their respective reservoir hosts are increasing constantly, and over 40 species and subspecies of *Bartonella* have now been detected in domestic and wild animals including cats, dogs, rodents, cattle, deer, bats, and so on^2^. Several *Bartonella* species were recognized as human pathogens, such as *B. bacilliformis*^3^*, B. quintana*^4^*, B. henselae*^5^*, B. elizabethae*^6^*, B. clarridgeiae*^7^*, B. koehlerae*^8^*, B. vinsonii* subsp*. arupensis*^9^*, B. vinsonii* subsp*. berkhoffii*^10^*, B. grahamii*^11,12^*, B. rochalimae*^13^*, B. tamiae*^14^*, B. ancashensis*^15^*, B. washoensis*^16^, and so on, which could cause endocarditis, myocarditis, neuroretinitis, meningitis, splenomegaly, lymphadenopathy and neurologic disorders in humans.

Small mammals**,** particularly rodents are considered natural reservoirs of many *Bartonella* species. Previous studies have reported that the infection rate of *Bartonella* is as high as 70% in rodents worldwide^17^, including in America^18^, Asia^19^, Africa^20^, and Europe^21^. Furthermore, many pathogenic *Bartonella* can be detected in rodents, which indicated that investigating the epidemiological characteristics of *Bartonella* in rodents is of great significance for the prevention and control of human Bartonellosis.

In the previous study, our team had detected *Bartonella* species in *Ochotona curzoiae* in the Qinghai-Tibet Plateau, with a positive rate of 18.99%^22^. However, few systematic investigations of *Bartonella* species in small mammals have been reported in the Qinghai-Tibet Plateau. The Qaidam Basin, located in the Haixi Mongolian Tibetan autonomous prefecture, northwest of Qinghai Province and northeastern Qinghai-Tibet Plateau, is the highest basin in China, with an altitude between 2600 and 3000 m; nearly 20 species of rodents have been reported to inhabit this area^23^. Furthermore, the Qaidam Basin, is a famous tourist attraction, attracting many visitors each year; therefore, the prevention and control of rodent-related pathogenic microorganisms are important for public health.

Accordingly, in this study, we investigated the prevalence and genetic diversity of *Bartonella* species in small mammals (including rodents and *Ochotona curzoiae*) from the Qaidam Basin. Our findings provided insights into the distribution of *Bartonella* in small mammals and the resulting public health threat in this region.

## Results

### Animal collection

**Figure 1:**
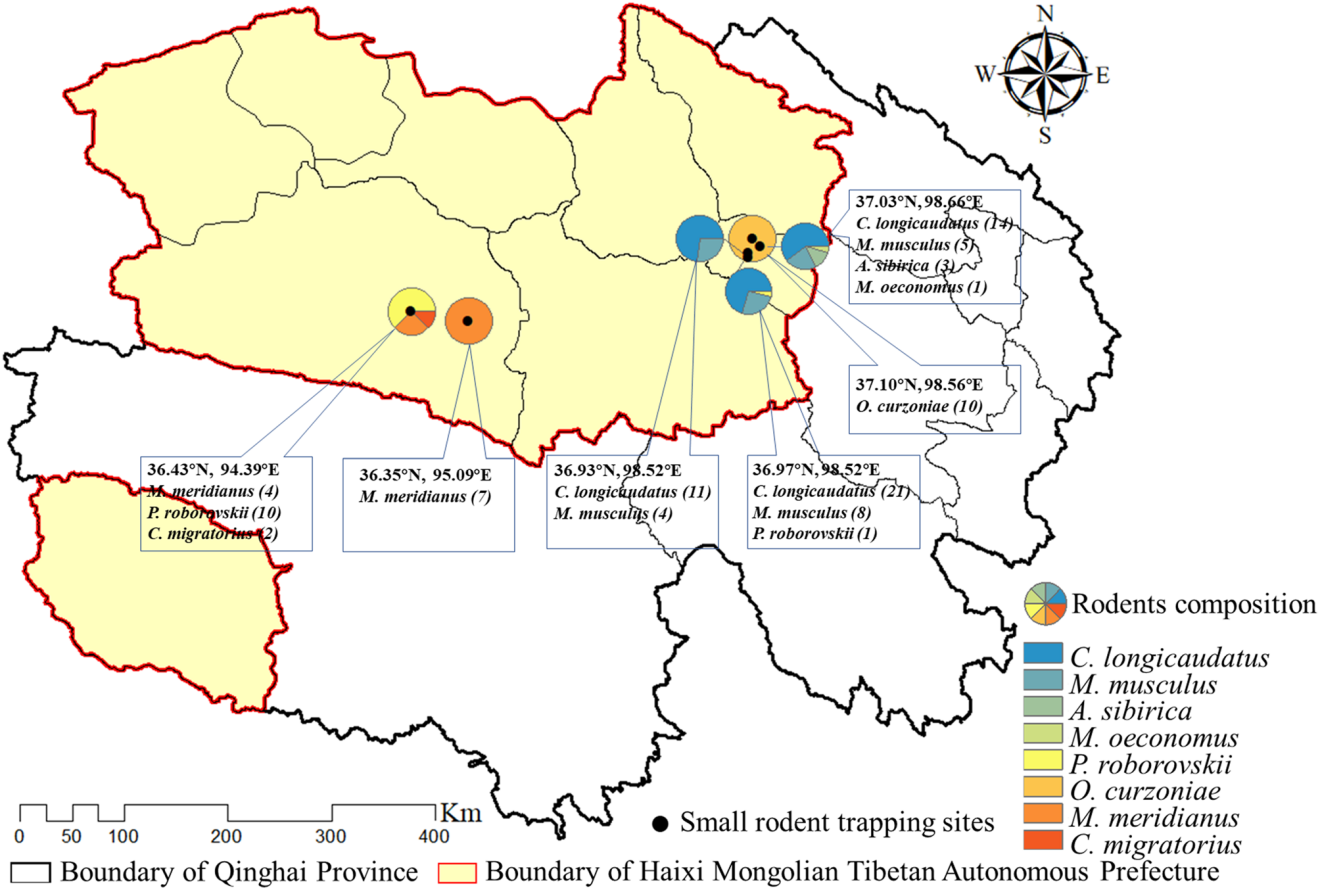
Geographical distribution of the trapped small mammals and the study areas consist of the sampling sites in two counties of Haixi Prefecture, China.

In total, 101 small mammals were captured from six trapping sites, including 78 in Wulan County (01-78 QHWL) and 23 in Ge’ermu City (79-101 QHGEM). All captured small mammals were identified morphologically into eight species, including *Cricetulus longicaudatus* (46), *Mus musculus* (17), *Phodopus roborovskii* (11), *Meriones meridians* (11), *Ochotona curzoniae* (10), *Allactaga sibirica* (3), *Cricetulus migratorius* (2) and *Microtus oeconomus* (1). The geographical distribution of the trapped small mammals was shown in Fig. 1.

### *Bartonella* infections

There were 97 spleens and 74 brains of 101 small mammals (excluded uncollected and contaminated specimens) used for *Bartonella* isolation, and the pure colonies obtained were then confirmed by polymerase chain reaction (PCR) amplification of partial *gltA* gene (379 bp)*.* In total, 39 small mammals, classified into four species (*Cricetulus longicaudatus* [29/46], *Microtus oeconomus* [1/1], *Allactaga sibirica* [3/3], *Meriones meridianus* [6/11]), were positive for *Bartonella* infection; 21 were positive in both the brain and spleen, 12 were positive in the spleen, and six were positive in the brain, with an overall infection rate of 38.61% (39/101). The positive rates in different tissues were not statistically significant (34.02% versus 36.49% for brain and spleen, respectively, *χ*^2^ = 0.112, *P* = 0.738) (Table 1). And the positive rate was 44.64% (25/56) in female and 31.11% (14/45) in male, which the difference was not statistically significant too (*χ*^2^ = 1.927, *P* = 0.165).

**Table 1:** Positive rate of *Bartonella* infection in different tissues of small mammals.

Host	Spleen			Brain			Total		
	No. cultivation	No. PCR positive	Positive rate (%)	No. cultivation	No. PCR positive	Positive rate (%)	No. captured	No. PCR positive	Positive rate (%)
CL	43	26	60.47	33	21	63.64	46	29	63.04
MuM	17	0	0.00	11	0	0.00	17	0	0.00
PR	10	0	0.00	10	0	0.00	11	0	0.00
MM	11	3	27.27	9	5	55.56	11	6	54.55
OC	10	0	0.00	9	0	0.00	10	0	0.00
AS	3	3	100.00	1	1	100.00	3	3	100.00
CM	2	0	0.00	1	0	0.00	2	0	0.00
MO	1	1	100.00	0	–	–	1	1	100.00
Total	97	33	34.02	74	27	36.49	101	39	38.61

CL, *Cricetulus longicaudatus*; MuM, *Mus musculus*; PR, *Phodopus roborovskii*; MM, *Meriones meridianus*; OC, *Ochotona curzoniae*; AS, *Allactaga sibirica*; CM, *Cricetulus migratorius*; MO, *Microtus oeconomus.*

There were 45 small mammals of three species captured in farmlands, with a *Bartonella* infection rate of 40.00% (18/45). Twenty small mammals of three species were captured in forests, with an infection rate of 60.00% (12/20). Additionally, 10 small mammals of one specie were captured in meadows, with no *Bartonella* infection, and 26 small mammals of four species were captured in semi-desert areas, with an infection rate of 34.62% (9/26). Thus, the infections rates in different habitats were significantly different (*χ*^2^ = 10.361, *P* = 0.016) (Table 2).

**Table 2:** Positive rate of *Bartonella* infection of small mammals in different habitats.

Habitats	Host								No. captured	No. PCR positive	Positive rate (%)
	CL	MuM	PR	MM	OC	AS	CM	MO			
Farmland	32	12	1	0	0	0	0	0	45	18	40.00
Forest	14	5	0	0	0	0	0	1	20	12	60.00
Meadow	0	0	0	0	10	0	0	0	10	0	0.00
Semi-desert	0	0	10	11	0	3	2	0	26	9	34.62
Total	46	17	11	11	10	3	2	1	101	39	38.61

### Identification of *Bartonella* species

Through BLAST analysis of the *gltA* gene, 40 *Bartonella* strains were obtained, including 18 strains isolated in spleens or brains of different small mammals, 20 strains isolated both in the spleens and brains of the same small mammals, and two strains isolated in the spleen and brain from one small mammal. Overall, 29 isolates from *Cricetulus longicaudatus* and one isolate from *Microtus oeconomus* were *B. grahamii* with 96.75–99.15% identity; three isolates from *Allactaga sibirica* were *B. jaculi* with 97.34–97.63% identity; four isolates from *Meriones meridianus* were Candidatus *B. gerbillinarum* with 92.06–96.06% identity; and three isolates from *Meriones meridianus* were *B. krasnovii* with 97.73–98.57% identity.

**Figure 2:**
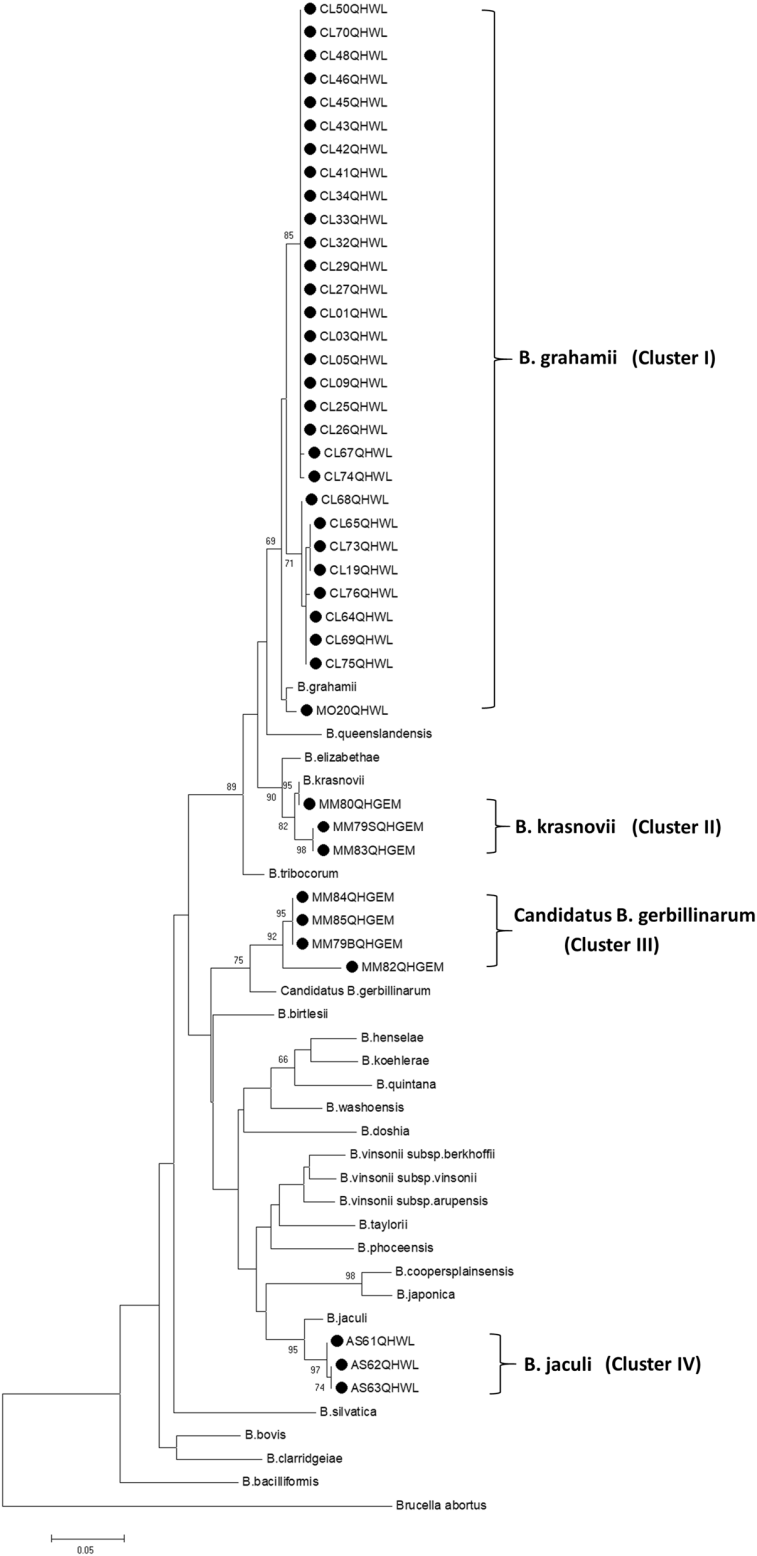
Phylogenetic trees constructed with concatenations of *gltA*, *ftsZ*, *rpoB* and *ribC* genes.

We used the maximum likelihood (ML), neighbor-joining (NJ), minimum-evolution (ME), and unweighted pair-group method with arithmetic mean to construct phylogenetic trees and obtained the same results; thus, the ML method was used for further analyses. Phylogenetic trees were constructed based on the DNA sequences of the concatenations of *gltA*, *ftsZ*, *rpoB* and *ribC* genes (2483 bp), and all isolates clustered into four clusters, i.e., clusters I to IV (Fig. 2). Strains belonging to cluster I were closely related to *B. grahamii*, strains belonging to cluster II were closely related to *B. krasnovii,* strains belonging to cluster III were closely related to Candidatus *B. gerbillinarum* and strains belonging to cluster IV were closely related to *B. jaculi.* In cluster III, MM82QHGEM was separated from the other three isolates by a long distance and showed 92.06% identity with reference Candidatus *B. gerbillinarum,* indicating that this isolate might be a new species of *Bartonella*^24^*.*

**Figure 3:**
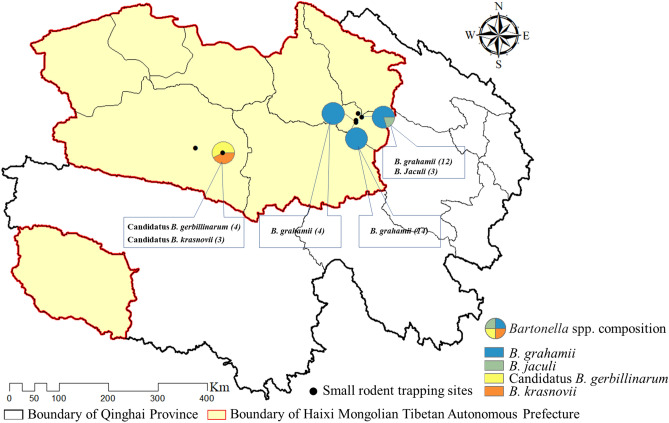
*Bartonella* species composition in different sampling sites in two counties of Haixi Prefecture, China.

Overall, our findings indicated that *B. grahamii*, *B. jaculi*, *B. krasnovi,* and Candidatus *B. gerbillinarum* were prevalent in the Qaidam Basin, and that *B. grahamii* was the dominant *Bartonella* species. *Bartonella* was not detected in the small mammals from two of six trapping sites, and the distribution of *Bartonella* species showed obvious geographical differences. Moreover, *B. grahamii* and *B. jaculi* were distributed in Wulan County, whereas Candidatus *B. gerbillinarum* and *B. krasnovii* were distributed in Ge’ermu City (Fig. 3).

Interestingly, our study also showed an association between *Bartonella* species and small mammal species. *B. grahamii* was specific for *Cricetulus longicaudatus*, *B. jaculi* was specific for *Allactaga sibirica*, and Candidatus *B. gerbillinarum* and *B. krasnovii* were specific for *Meriones meridianus*. In addition, two *Bartonella* species, i.e., Candidatus *B. gerbillinarum* and *B. krasnovii* were isolated from different tissues in one *Meriones meridianu*. Candidatus *B. gerbillinarum* was isolated from the brain, and *B. krasnovii* was isolated from the spleen, suggesting that one rodent could carry more than one *Bartonella* species (Table 3). It indicated the coinfection phenomenon existed in Bartonella species, which was consistent with previous studies^25,26^.

**Table 3:** Distribution of *Bartonella* infection in different small mammals.

Host	n	*B. grahamii* (%)	*B. jaculi* (%)	Candidatus *B. gerbillinarum* (%)	*B. krasnovii* (%)
CL	46	29 (63.04)	0 (0.00)	0 (0.00)	0 (0.00)
MuM	17	0 (0.00)	0 (0.00)	0 (0.00)	0 (0.00)
PR	11	0 (0.00)	0 (0.00)	0 (0.00)	0 (0.00)
MM	11	0 (0.00)	0 (0.00)	4 (36.36)	3 (27.27)
OC	10	0 (0.00)	0 (0.00)	0 (0.00)	0 (0.00)
AS	3	0 (0.00)	3 (100.00)	0 (0.00)	0 (0.00)
CM	2	0 (0.00)	0 (0.00)	0 (0.00)	0 (0.00)
MO	1	1 (100.00)	0 (0.00)	0 (0.00)	0 (0.00)
Total	101	30 (29.70)	3 (2.97)	4 (39.60)	3 (2.97)

Two *bartonella* species were detected in MM79QHGEM, *B. krasnovii* detected in spleen, and Candidatus *B. gerbillinarum* detected in brain.

### Phylogenetic analysis

Phylogenetic analysis based on *gltA* sequences showed that *B. grahamii* in the Qaidam Basin was mainly clustered into three clusters. Some strains from *Cricetulus longicaudatus* clustered with *B. grahamii* from *Apodemus speciosus* in Japan, some strains from *Cricetulus longicaudatus* clustered with *B. grahamii* from *Ochotona curzoniae* in China, and one strain from *Microtus oeconomus* was clustered separately, indicating the genetic diversity of *B. grahamii* prevalent in the Qaidam Basin.

**Figure 4:**
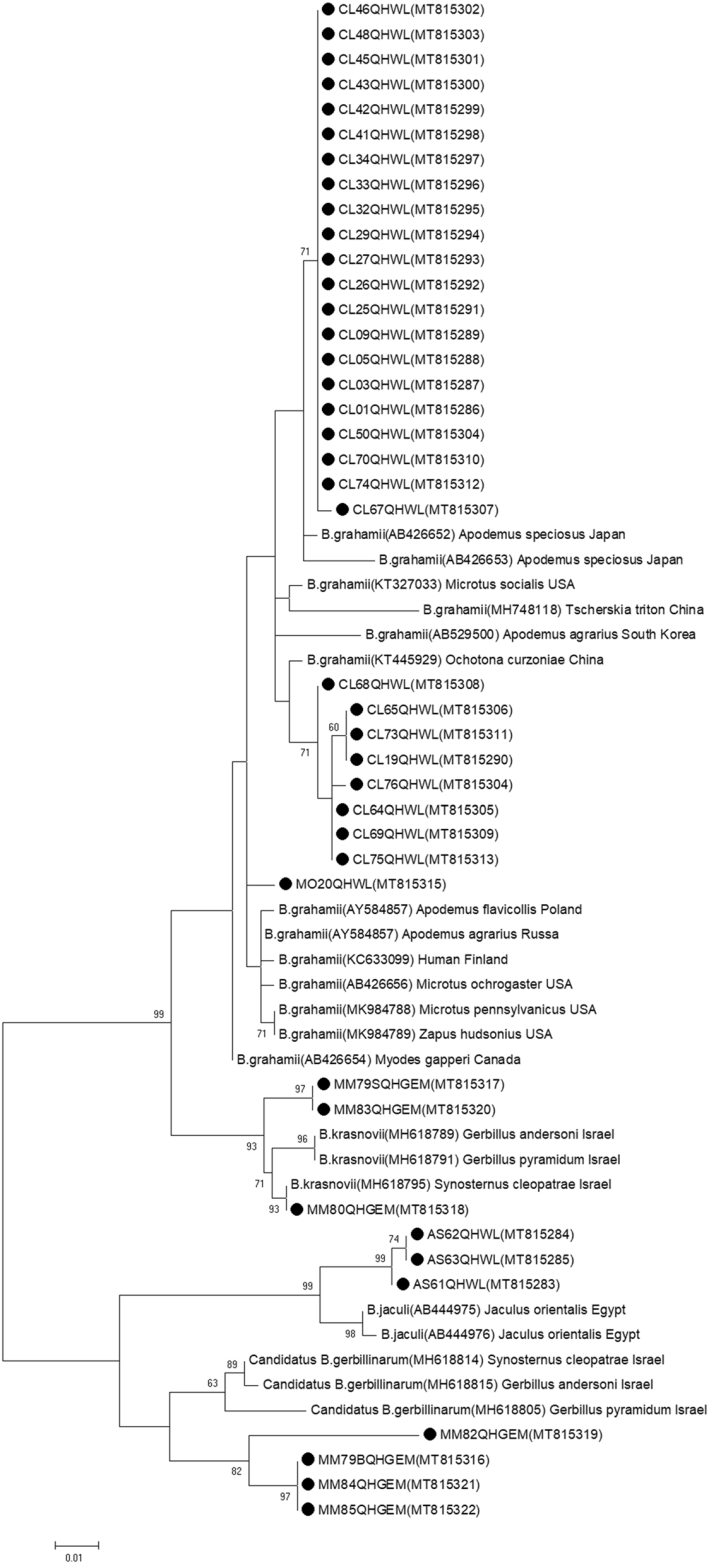
Phylogenetic analysis based on *gltA* gene.

Three strains of AS61QHWL-AS63QHWL from *Allactaga sibirica* were clustered with *B. jaculi* from *Jaculus orientails* in Egypt, four strains of MM79BQHGEM, MM82QHGEM, MM84QHGEM and MM85QHGEM from *Meriones meridianus* were clustered with Candidatus *B. gerbillinarum* from *Synosternus cleopatrae*, *Gerbillus andersoni* and *Gerbillus pyramidum* in Israel, and three strains of MM79SQHGEM, MM80QHGEM, and MM83QHGEM from *Meriones meridianus* were clustered with *B. krasnovii* from *Synosternus cleopatrae*, *Gerbillus andersoni* and *Gerbillus pyramidum* in Israel. The results showed that the Qaidam Basin strains of *B. jaculi,* Candidatus *B. gerbillinarum* and *B. krasnovii* were clustered with the relative reference strains, but formed distinct branches, except MM80QHGEM. Thus, these findings suggested that *Bartonella* infection was rodent specific and continued to evolve (Fig. 4).

## Discussion

*Bartonella* species are highly prevalent in small mammals worldwide, which have the close contact with humans. It is of great significance to investigate the epidemiological and ecological characteristics of *Bartonella* infection in small mammals from different areas. In this study, we observed the prevalence and genetic diversity of *Bartonella* species in small mammals in the Qaidam Basin. The infection rate of *Bartonella* species in small mammals was 38.61%, which was higher than that of 18.99% in *Ochotona curzoniae* in our previous study^22^, and higher than that in most areas of China^27^, but lower than that in many other countries, including Russia (60–83%), Canada (48–90%), Netherlands (72%), etc^17^. In addition, the infection rate was significantly different in different habitats (farmlands, forests, meadows, and semi-desert areas), with the highest infection rates observed in forests.

The nutritional requirements of *Bartonella* make it difficult to culture in vitro^28^. Generally, the spleen tissue is used in Bartonella culture. In this study, brain tissue was successfully used for *Bartonella* isolation for the first time. Additionally, we found that the positive rates in different tissues (spleens and brains) of small mammals did not differ significantly. Moreover, we detected two *Bartonella* species in the same small mammal, i.e., *B. krasnovii* in the spleen and Candidatus *B. gerbillinarum* in the brain, indicating the complexity of *Bartonella* infection in small mammals. A previous study indicated that there might be a high *Bartonella* coinfection rate in rodents^29^. Therefore, in order to further explore the coinfection of *Bartonella* species, additional studies of multi-tissue culture, multi-clone detection, and multiple PCR detection by using well-defined species or genotype PCR primer sets are needed^25,26,29^.

Several rodent-associated *Bartonella* species have been implicated as the causative agents of human disease. Here, we obtained four *Bartonella* species in rodents, *B. grahamii*, *B. jaculi*, *B. krasnovii* and Candidatus *B. gerbillinarum*. Especially, *B. grahamii* was detected in *Cricetulus longicaudatus* specificly, *B. jaculi* was detected in *Allactaga sibirica* specificly, Candidatus *B. gerbillinarum and B. krasnovii* were detected in *Meriones meridianus* specificly, indicating rodent-specific characteristics. *B. grahamii* was the dominant epidemic strain circulating in the Qaidam Basin, which was associated with neuroretinitis and cat scratch disease (CSD) in immunocompromised people^11,12^, suggesting that *Bartonella* species detected in *Cricetulus longicaudatus* may have the ability to cause human disease. It was reported that *Bartonella* species antibodies and DNA were detected in cerebrospinal fluid of cats and dogs^30,31^, suggesting the possible relationship between *Bartonella* infection and central nervous system disease. *Bartonella* isolation from the rodent brain supported this observation. Until now, *B. henselae* and *B. quintana* were reported to cause central nervous system infection^32,33^, however, the effects of *B. grahamii* on the central nervous system needs further investigation.

Through phylogenetic analysis based on *gltA* sequences collected from the GenBank, *B. grahamii* from *Cricetulus longicaudatus* in the Qaidam Basin was mainly clustered with *B. grahamii* from *Apodemus speciosus* in Japan^34^ and *Ochotona curzoniae* in China^22^. Some *B. grahamii* prevalent in the Qaidam Basin might have high homology with strains from Japan, which was consistent with the previous study^34^. *B. jaculi,* Candidatus *B. gerbillinarum* and *B. krasnovii* have only been reported in Eygpt^35^ and Israel^29^, which pathogenicity was not clear and needs further study. However, in this study, *Bartonella* infection was not detected in *Ochotona curzoniae*, possibly because of the differences in sampling sites and the small number of samples collected, which need further investigation.

## Conclusions

This study provided a better understanding of the prevalence and genetic diversity of *Bartonella* species in small mammals from the Qaidam Basin. Four *Bartonella* species were detected in rodents, among which *B. grahamii* was the dominant strain, and potentially pathogenic to humans. Additionally, *Bartonella* were isolated from rodent brains for the first time, and two *Bartonella* species were detected in different tissues of the same rodent, which indicated the complexity of *Bartonella* infection and the necessity for multi-tissue culture, multi-clone detection and multiple PCR detection. Our results raise the potential threats to public health by the *Bartonella* species, and surveillance of *Bartonella* in animals and investigation of suspected clinical cases in humans need to be strengthened in the Qaidam Basin.

## Materials and methods

### Ethical statement

This study was approved by the Ethics Committee of Chinese Center for Disease Control and Prevention (No: ICDC-2015001). Small mammals were sampled with the help of Wulan Center for Disease Control and Prevention, and Ge’ermu Center for Disease Control and Prevention. All animals were treated according to the Guidelines of Regulations for the Administration of Laboratory Animals (Decree No. 2 of the State Science and Technology Commission of the People's Republic of China, 1988) and the Guidelines for Treating Animals Kindly from Ministry of Science and Technology of the People’s Republic of China. All efforts were made to minimize discomfort to the animals.

### Sample collection

According to the previous study^36^, small mammals were captured using snap traps in a diversity of habitats in July 2018 in Wulan County (latitude 36.32°–37.33°N, longitude 97.02°–99.45°E) and Ge’ermu City (latitude 35.18°–37.80°N, longitude 91.72°–95.85°E), Qinghai Province. And the small mammal species were determined morphologically. The spleens and brains were harvested under sterile conditions from each animal after euthanasia, and all samples were stored in liquid nitrogen until use.

### Bartonella culture

Approximately 20 mg of each spleen and brain sample from the small mammal was homogenized by adding 200 μL sterilized trypsin soy broth (BD Biosciences, Franklin Lakes, NJ, USA), plated onto two trypsin soy agars containing 5% (vol/vol) defiber sheep blood (BD Biosciences), and incubated at 37 °C in an atmosphere containing 5% CO_2_. Pure colonies of *Bartonella* species were obtained according to previous methods^22^.

### DNA extraction, PCR amplification and DNA sequencing

Crude DNA was extracted according to the previous method^22^. PCR was performed to detect the *Bartonella* citrate synthase (*gltA*) gene. Then, *gltA* positive strains were evaluated for amplification of *ftsZ*, RNA polymerase beta subunit (*rpoB*) and *ribC* genes. DNA amplification was performed in 50 μL mixtures containing 25 μL 2 × TransTaq-T PCR SuperMix (Beijing TransGen Biotech Co., Ltd., Beijing, China), 22 μL double-distilled H_2_O, 1 μL (10 μmol/L) of each primer (sequences listed in Table 4), and 1 μL of DNA template. *gltA* amplification was performed under the following conditions: one cycle for 5 min at 94 °C; 33 cycles for 30 s at 94 °C, 30 s at 53 °C, and 20 s at 72 °C; and a final extension for 7 min at 72 °C. The annealing temperatures for amplification of the *ftsZ*, *rpoB* and *ribC* were 55 °C, 50 °C and 50 °C, respectively. Next, 5 μL of each PCR product was run on 1% agarose gels, stained with ethidium bromide, and visualized using a gel imaging system (Bio-Rad, Hercules, CA, USA). The expected PCR products were purified using the QIAquick PCR Purification Kit (Qiagen, Hilden, Germany) according to the manufacturer’s protocols, and then sequenced using specific primers for *gltA*, *ftsZ*, *rpoB* and *ribC* with an Applied Biosystems 3730 xl Genetic Analyzer (Applied Biosystems, Foster City, CA, USA).

**Table 4:** The primers used in this study.

Gene	Primer	Primer sequence (5′–3′)	Product length (bp)
*gltA*	BhCS781.p	GGGGACCAGCTCATGGTGG	379
	BhCS1137.n	AATGCAAAAAGAACAGTAAACA^37^	
*ftsZ*	Bfp1	ATTAATCTGCAYCGGCCAGA	896
	Bfp2	ACVGADACACGAATAACACC^38^	
*rpoB*	1400F	CGCATTGGCTTACTTCGTATG	866
	2300R	GTAGACTGATTAAACGCTG^39^	
*ribC*	BARTON-1	TAACCGATATTGGTTGTGTTGAAG	577
	BARTON-2	TAAAGCTAGAAAGTCTGGCAACATAACG^40^	

### Phylogenetic analysis

The sequences generated in this study were submitted to the GenBank (accession numbers MT815283-MT815438). The nucleotide sequence homology was blasted against reported *Bartonella* species sequences in the GenBank using the BLAST program at the National Center for Biotechnology Information Website (http://blast.ncbi.nlm.nih.gov/Blast.cgi). Phylogenetic tree was created using the maximum-likelihood method with MEGA version 7.0, and bootstrap values were calculated with 1000 replicates^41,42^ (incomplete and poor quality sequences were excluded from phylogenetic analysis). *Brucella abortus* was used as the outgroup.

### Statistical analysis

The positive rates of *Bartonella* in different habitats, genders and tissues of small mammals were analyzed using the Chi-square test. All data were analyzed using SPSS 22.0 (SPSS, Inc., Chicago, IL, USA). *P* < 0.05 was considered statistically significant.

### Consent to publish

All the authors consent to publish the article in its present form.

## Data Availability

The data supporting the conclusions of this article are included within the article.
